# Supplementary material for: Administrative-driven hierarchical management of atrial fibrillation on cardiovascular events: a prospective matched cohort study
Source: Nat Commun. 2025 Dec 16;16:11395. doi: 10.1038/s41467-025-66203-y (PMC12738700; doi:10.1038/s41467-025-66203-y)
Supplement: Supplementary file 1 — Supplementary Information [file 41467_2025_66203_MOESM1_ESM.pdf]

Administrative-driven hierarchical management of atrial fibrillation on cardiovascular events: a prospective matched cohort study

Mu Chen, MD; Mingzhe Zhao, MD; Yuli Yang, MD; Xin Cui, MSc; Chunfang Wang, MSc; Tian Xia, MSc; Wenqi Tian, MSs; Peng Liao, PhD; Yudong Fei, PhD; Peng-Cheng Yao, MD; Xiaoxiao Zhu, MSc ; Yongbo Wu, MSc; Mei Yang, MD; Jian Sun, MD; Li Luo, PhD; Hong Wu, MD; Qunshan Wang, MD*; Yi-Gang Li, MD*

Supplementary information

Supplementary Fig. 1. Scheme of the ADHM model of AF.

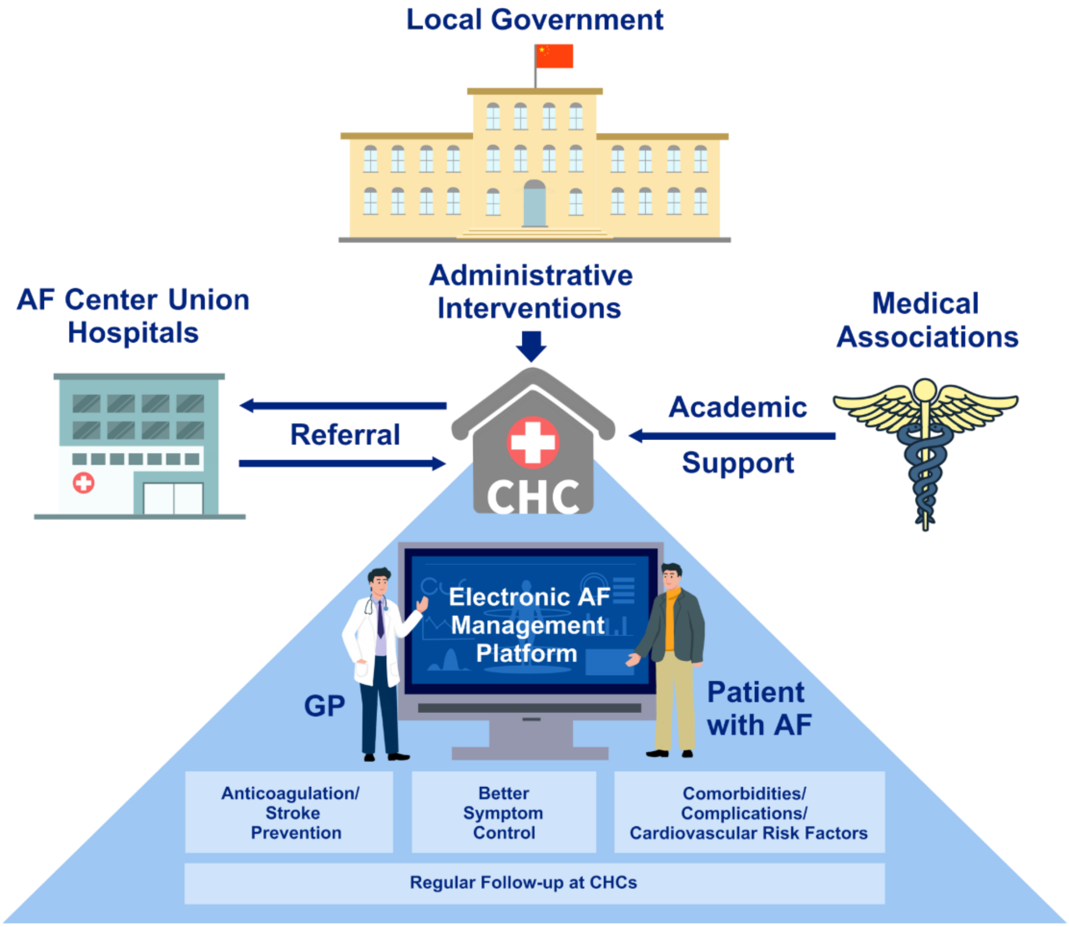

ADHM= administrative-driven hierarchical management; AF=atrial fibrillation; CHC=community healthcare center; GP=general practitioner

Supplementary Fig. 2. Outcomes of patients with AF before/during and after the shift in China's COVID-19 policies.

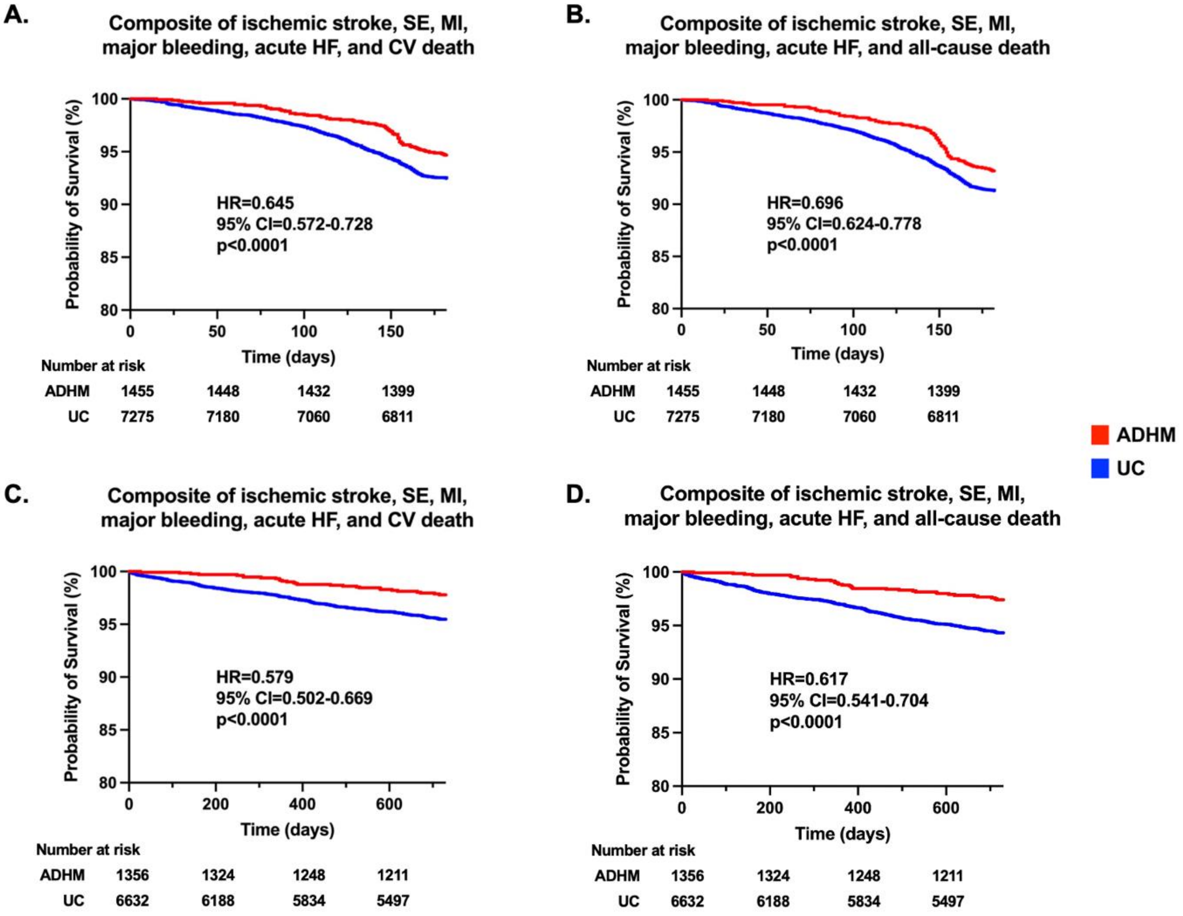

Shown are Kaplan-Meier estimates of the composite of CV death, ischemic stroke, SE, MI, major bleeding, and acute HF (primary endpoint), and the composite of all-cause death, ischemic stroke, SE, MI, major bleeding, and acute HF, before (A, B) and after (C, D) January 31, 2023. ADHM= administrative-driven hierarchical management; AF= atrial fibrillation; CI= confidence interval; CV= cardiovascular; HF=heart failure; HR= hazard ratio; MI= myocardial infarction; TIA= transient ischemic attack; SE= systemic embolism; UC=usual care. The log-rank test and presented as HR and 95% CI. Two-sided P value was adopted.

Supplementary Fig. 3. Other secondary outcomes of patients with AF.

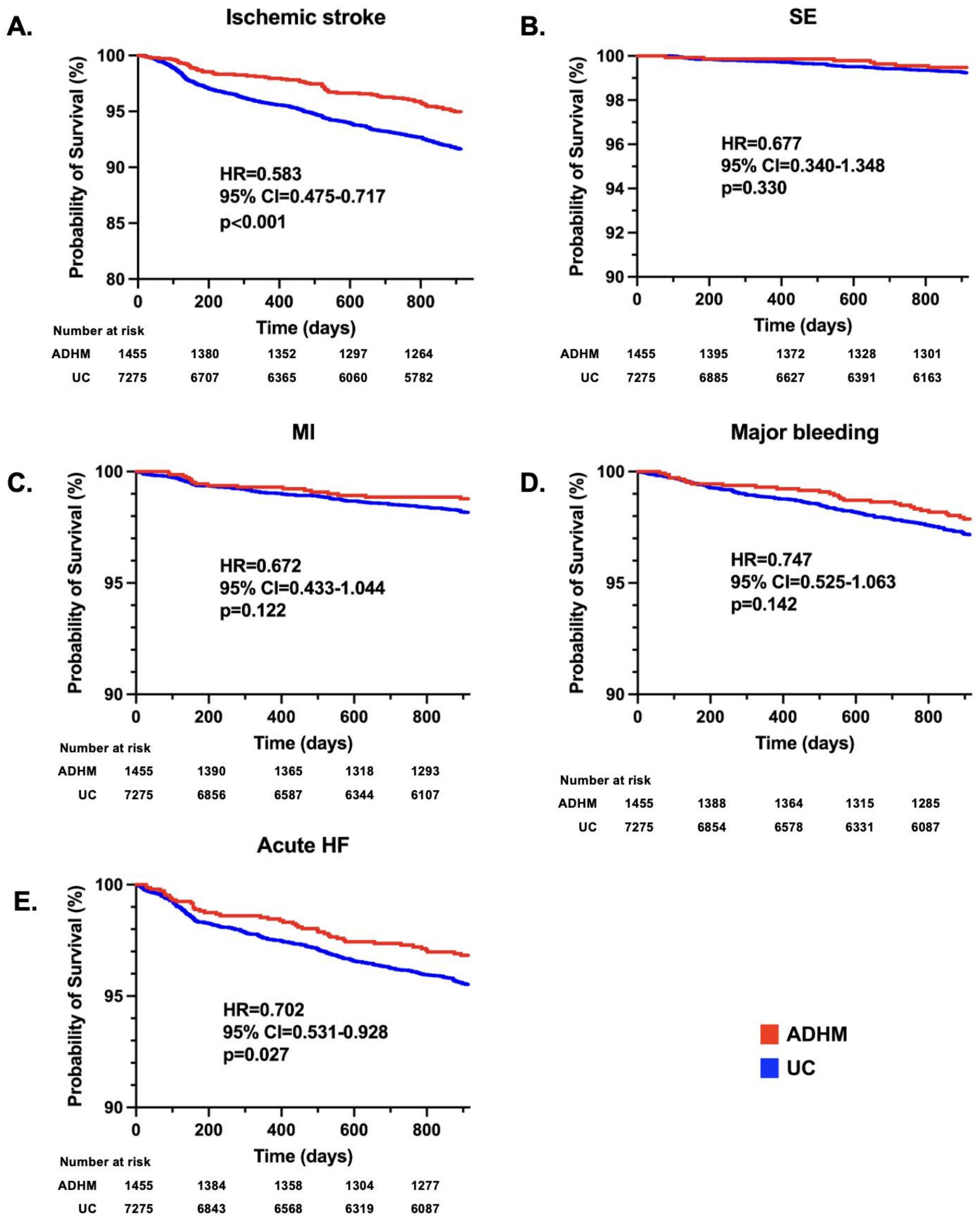

Shown are Kaplan-Meier estimates of the secondary endpoint events, including ischemic stroke (A), SE (B), MI (C), major bleeding (D), and acute HF (E) in the ADHM cohort (red curve) and the UC cohort (blue curve), respectively. ADHM= administrative-driven

hierarchical management; AF= atrial fibrillation; CI= confidence interval; HF=heart failure; HR= hazard ratio; MI= myocardial infarction; TIA= transient ischemic attack; SE= systemic embolism; UC=usual care. The log-rank test and presented as HR and 95% CI. Two-sided P value was adopted.

Supplementary Fig. 4. Scheme of the Shanghai AF management platform.

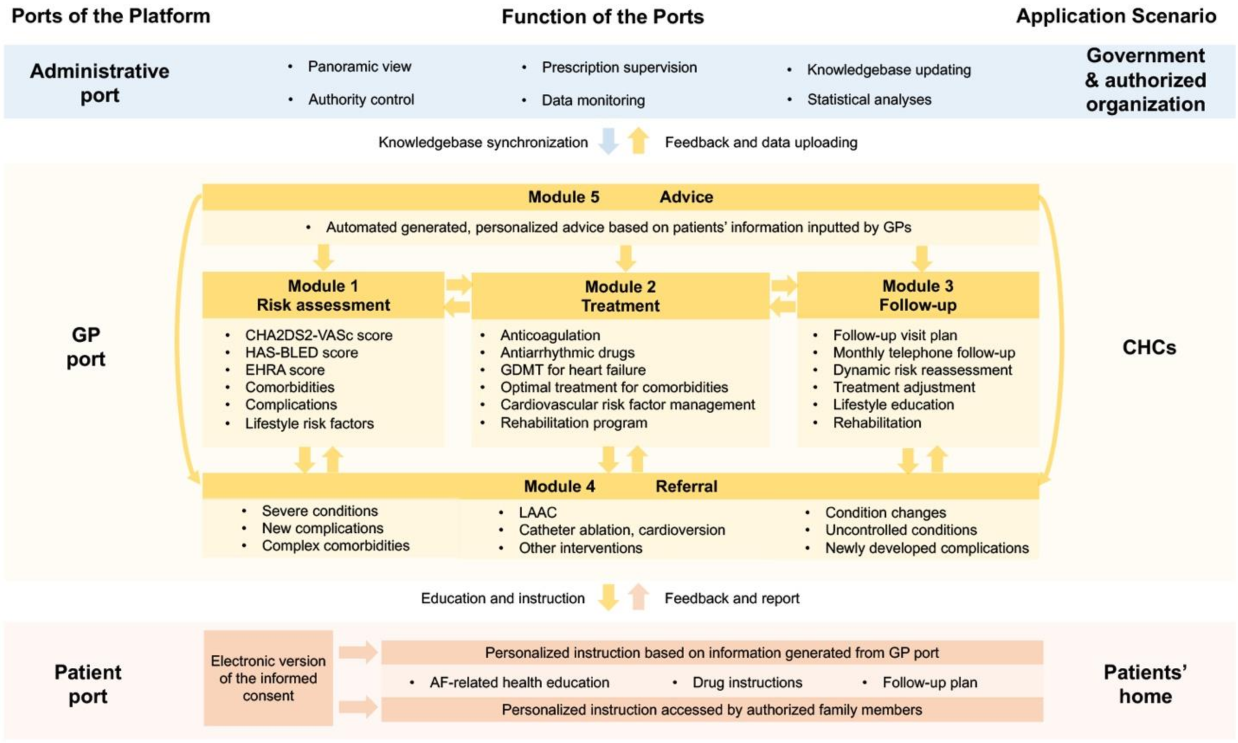

The content, function, and application scenario of the three ports of the electronic Shanghai AF management platform, including administrative, GP, and patient ports.

AF= atrial fibrillation; CHC=community healthcare center; GDMT= guideline-directed medical therapy; GP= general practitioner; LAAC= left atrial appendage closure.

Supplementary Fig. 5. Screening capture (and translation) of the GP port of the Shanghai AF Management Platform.

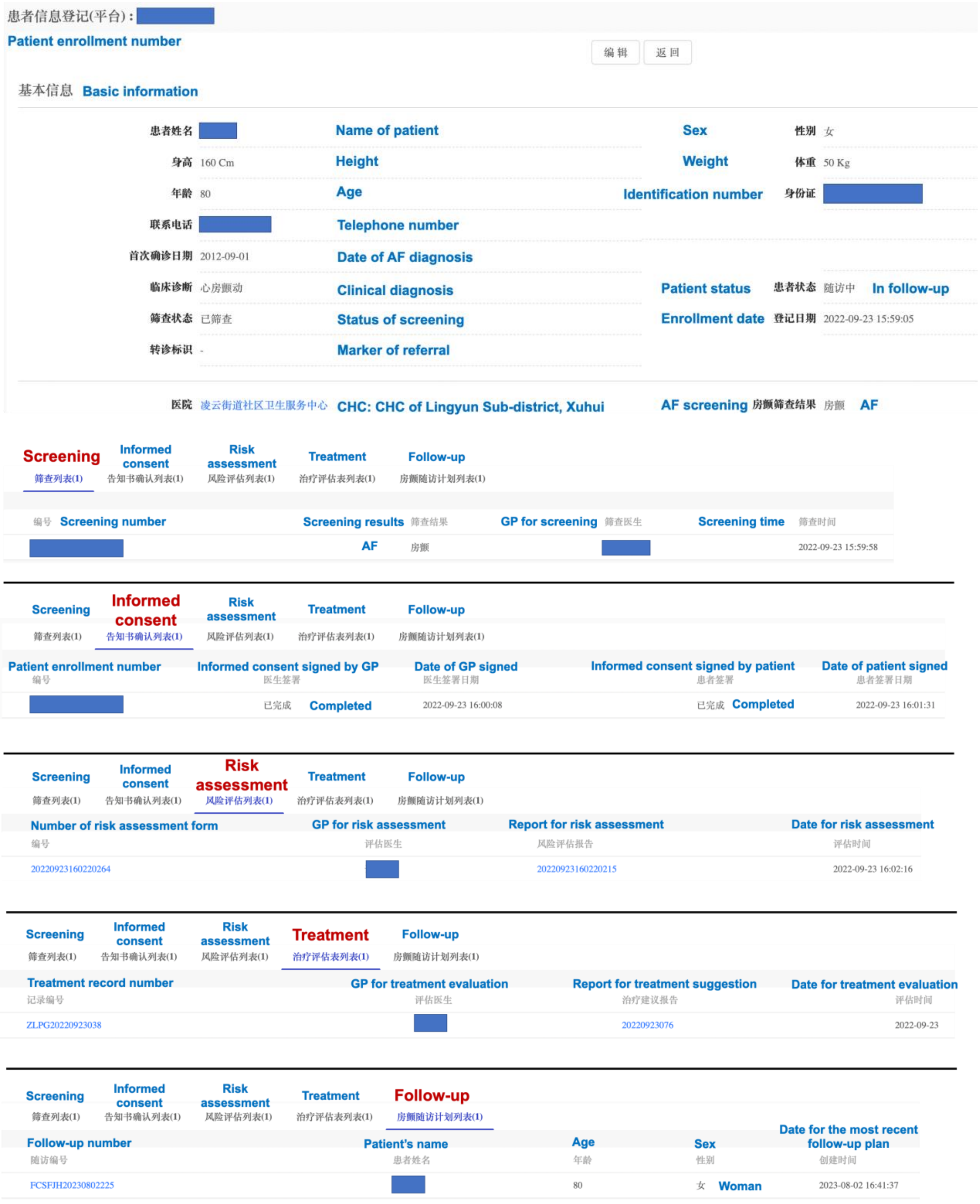

Supplementary Fig. 6. Example (Screening capture and translation) of the risk assessment and therapeutic suggestions automatedly generated by the Shanghai AF Management Platform.

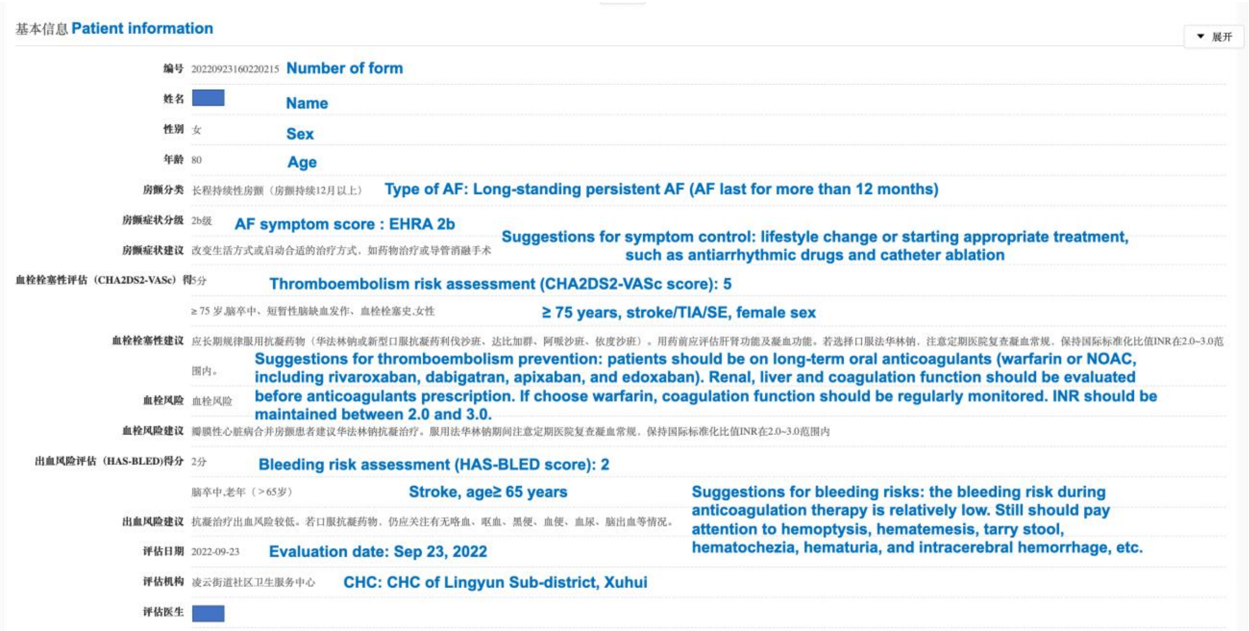

Supplementary Fig. 7. Events detected by GP follow-up versus database screening in the ADHM cohort in the initial 180 days.

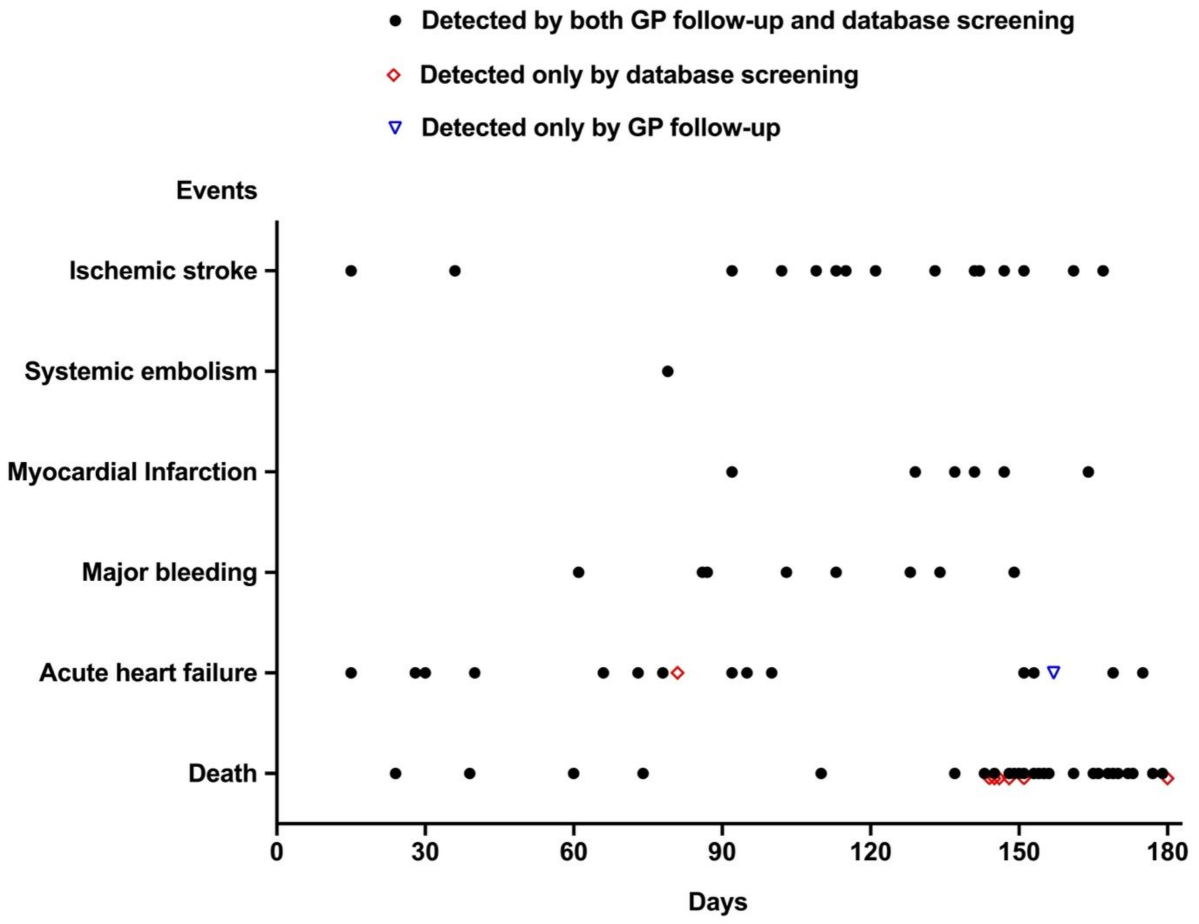

Plots indicated the outcome events, including ischemic stroke, systemic embolism, myocardial infarction, major bleeding, acute heart failure, and death, detected by the methods of GP follow-up and/or database screening in the ADHM cohort in the initial 180 days. ADHM= administrative-driven hierarchical management; GP= general practitioner.

Supplementary Table 1. Predictors of the primary outcome in patients in both ADHM and UC groups.

Variables	Univariate HR (95% CI)	P value	Multivariate HR (95% CI)	P value
Baseline characteristics				
Age (years)	1.059 (1.052-1.065)	<0.001	1.053 (1.041-1.065)	<0.001
Female sex	1.286 (1.161-1.424)	<0.001	1.203 (0.972-1.490)	0.090
Hypertension	2.457 (1.894-3.188)	<0.001	1.176 (0.788-1.754)	0.427
Diabetes	1.381 (1.245-1.532)	<0.001	1.212 (0.974-1.509)	0.085
History of stroke/TIA	2.325 (2.088-2.588)	<0.001	1.779 (1.212-2.611)	0.003
History of SE	1.548 (1.128-1.885)	0.004	1.099 (0.839-1.440)	0.493
History of heart failure	1.793 (1.614-1.992)	<0.001	1.452 (1.163-1.812)	0.001
Periphery artery disease	1.690 (1.320-2.163)	<0.001	1.379 (1.065-1.787)	0.015
History of MI	1.458 (1.112-1.911)	0.006	1.392 (0.991-1.955)	0.057
Valvular heart disease	0.852 (0.647-1.120)	0.251		
COPD	1.289 (1.145-1.451)	<0.001	0.923 (0.813-1.048)	0.217
History of major bleeding	1.399 (1.202-1.629)	<0.001	0.940 (0.656-1.347)	0.738
History of cancer	0.953 (0.815-1.115)	0.549		
History of renal deficiency	1.971 (1.609-2.415)	<0.001	1.327 (0.899-1.959)	0.154
History of liver deficiency	1.556 (1.367-1.771)	<0.001	1.199 (0.843-1.705)	0.313
CHA2DS2-VASc score	1.355 (1.315-1.396)	<0.001	0.907 (0.752-1.093)	0.306
HAS-BLED score	1.658 (1.569-1.752)	<0.001	1.164 (0.842-1.609)	0.359
During follow-up				
UC compared to ADHM	1.674 (1.435-1.952)	<0.001	1.838 (1.556-2.173)	<0.001
Regular anticoagulant prescription	0.872 (0.788-0.966)	0.009	0.962 (0.862-1.073)	0.487
Antiarrhythmic drug prescription	0.998 (0.899-1.108)	0.966		
Rate control drug prescription	1.028 (0.921-1.148)	0.616		
Statin prescription	0.975 (0.838-1.135)	0.747		
RAASi prescription	1.024 (0.912-1.151)	0.685		
MRA prescription	1.075 (0.917-1.262)	0.372		
SGLT2i prescription	1.058 (0.832-1.346)	0.643		
Catheter ablation	1.173 (0.928-1.483)	0.181		
LAAC	1.347 (0.942-1.926)	0.102		
Visit CHCs≥4 times per year	0.912 (0.824-1.011)	0.078	1.246 (1.113-1.396)	<0.001
Times of visit to higher level hospitals	1.005 (0.984-1.026)	0.645		

ADHM= administrative-driven hierarchical management; CHC= community healthcare center; COPD= chronic obstructive pulmonary disease; LAAC= left atrial appendage closure; MI= myocardial infarction; MRA= mineralocorticoid receptor antagonist; SE= systemic embolism; SGLT2i= sodium-glucose co-transporter 2 inhibitor; TIA= transient ischemic attack; UC= usual care. Univariate and multivariate analyses of predictors of the primary outcome were assessed with the employment of a Cox hazard regression model. Two-sided p value was adopted.

Supplementary Table 2. Predictors of the primary outcome in patients in the ADHM group.

Variables	Univariate		Multivariate	
	HR (95% CI)	P value	HR (95% CI)	P value
Baseline characteristics				
Age (years)	1.099 (1.079-1.120)	<0.001	1.062 (1.028-1.097)	<0.001
Female sex	1.426 (1.069-1.902)	0.016	0.733 (0.379-1.419)	0.357
Hypertension	4.223 (1.703-10.469)	0.002	2.922 (0.777-10.985)	0.112
Diabetes	1.572 (1.167-2.117)	0.003	0.695 (0.351-1.378)	0.298
History of stroke/TIA	2.561 (1.877-3.494)	<0.001	1.454 (0.465-4.546)	0.520
History of SE	1.089 (0.508-2.332)	0.827		
History of heart failure	2.903 (2.111-3.992)	<0.001	1.010 (0.511-1.996)	0.978
Periphery artery disease	1.089 (0.545-2.178)	0.808		
History of MI	2.104 (1.097-3.034)	0.025	0.904 (0.351-2.329)	0.834
Valvular heart disease	1.395 (0.688-2.827)	0.356		
COPD	1.790 (1.305-2.453)	<0.001	1.102 (0.778-1.561)	0.585
History of major bleeding	1.712 (1.156-2.536)	0.007	3.199 (1.005-10.184)	0.049
History of cancer	1.325 (0.880-1.993)	0.177		
History of renal deficiency	1.740 (1.020-2.966)	0.042	3.353 (0.980-11.469)	0.054
History of liver deficiency	1.615 (1.131-2.307)	0.008	3.624 (1.122-11.701)	0.031
CHA2DS2-VASc score	1.533 (1.400-1.677)	<0.001	1.589 (0.887-2.847)	0.119
HAS-BLED score	1.752 (1.504-2.041)	<0.001	0.401 (0.134-1.200)	0.102
During follow-up				
Regular anticoagulant prescription	0.592 (0.429-0.816)	0.001	0.555 (0.384-0.801)	0.002
Antiarrhythmic drug prescription	1.012 (0.759-1.350)	0.935		
Rate control drug prescription	1.439 (1.070-1.936)	0.016	1.217 (0.874-1.695)	0.244
Statin prescription	1.515 (1.116-2.057)	0.747		
RAASi prescription	1.628 (1.219-1.173)	0.001	1.354 (0.984-1.864)	0.062
MRA prescription	2.465 (1.796-3.383)	<0.001	1.289 (0.899-1.848)	0.167
SGLT2i prescription	1.457 (0.937-2.265)	0.095	1.407 (0.848-2.334)	0.186
Catheter ablation	0.491 (0.288-0.837)	0.009	0.830 (0.468-1.470)	0.522
LAAC	1.403 (0.593-3.323)	0.441		
Visit CHCs≥4 times per year	0.576 (0.420-0.791)	0.001	0.971 (0.709-1.329)	0.855
Times of visit to higher level hospitals	0.951 (0.869-1.040)	0.269		

ADHM= administrative-driven hierarchical management; CHC= community healthcare center; COPD= chronic obstructive pulmonary disease; LAAC= left atrial appendage closure; MI= myocardial infarction; MRA= mineralocorticoid receptor antagonist; SE= systemic embolism; SGLT2i= sodium-glucose co-transporter 2 inhibitor; TIA= transient ischemic attack; UC= usual care. Univariate and multivariate analyses of predictors of the primary outcome were assessed with the employment of a Cox hazard regression model. Two-sided p value was adopted.

Supplementary Table 3. Predictors of the primary outcome in patients in the UC group.

Variables	Univariate HR (95% CI)	P value	Multivariate HR (95% CI)	P value
Baseline characteristics				
Age (years)	1.054 (1.047-1.160)	<0.001	1.046 (1.034-1.059)	<0.001
Female sex	1.269 (1.137-1.415)	<0.001	1.248 (0.994-1.566)	0.056
Hypertension	2.291 (1.744-3.011)	<0.001	1.016 (0.666-1.550)	0.940
Diabetes	1.359 (1.216-1.519)	<0.001	1.232 (0.976-1.555)	0.079
History of stroke/TIA	2.306 (2.056-2.587)	<0.001	1.634 (1.087-2.457)	0.018
History of SE	1.591 (1.208-2.097)	0.001		
History of heart failure	1.683 (1.505-1.883)	<0.001	1.010 (0.511-1.996)	0.978
Periphery artery disease	1.869 (1.429-2.444)	<0.001	1.454 (1.101-1.921)	0.008
History of MI	1.376 (1.022-1.853)	0.036	1.323 (0.916-1.911)	0.135
Valvular heart disease	1.148 (0.852-1.547)	0.363		
COPD	1.228 (1.080-1.396)	0.002	0.892 (0.778-1.022)	0.100
History of major bleeding	1.365 (1.157-1.610)	<0.001	0.783 (0.536-1.145)	0.208
History of cancer	1.106 (0.934-1.310)	0.244		
History of renal deficiency	2.051 (1.644-2.559)	<0.001	1.164 (0.770-1.759)	0.473
History of liver deficiency	1.550 (1.348-1.781)	<0.001	1.044 (0.721-1.512)	0.821
CHA2DS2-VASc score	1.335 (1.294-1.378)	<0.001	0.885 (0.725-1.079)	0.228
HAS-BLED score	1.652 (1.557-1.753)	<0.001	1.347 (0.959-1.893)	0.086
During follow-up				
Regular anticoagulant prescription	0.994 (0.891-1.110)	0.919		
Antiarrhythmic drug prescription	1.023 (0.914-1.145)	0.694		
Rate control drug prescription	1.085 (0.959-1.227)	0.195		
Statin prescription	0.964 (0.804-1.156)	0.692		
RAASi prescription	11.023 (0.897-1.167)	0.734		
MRA prescription	0.907 (0.749-1.099)	0.318		
SGLT2i prescription	1.064 (0.795-1.423)	0.678		
Catheter ablation	1.190 (0.288-0.837)	0.009	1.082 (0.801-1.462)	0.607
LAAC	1.830 (1.216-2.754)	0.004	1.914 (1.211-3.023)	0.005
Visit CHCs≥4 times per year	1.037 (0.930-1.157)	0.511		
Times of visit to higher level hospitals	0.989 (0.967-1.011)	0.320		

CHC= community healthcare center; COPD= chronic obstructive pulmonary disease; LAAC= left atrial appendage closure; MI= myocardial infarction; MRA= mineralocorticoid receptor antagonist; SE= systemic embolism; SGLT2i= sodium-glucose co-transporter 2 inhibitor; TIA= transient ischemic attack; UC= usual care. Univariate and multivariate analyses of predictors of the primary outcome were assessed with the employment of a Cox hazard regression model. Two-sided p value was adopted.

Supplementary Table 4. Total medical costs per patient during the entire follow-up.

Cost per patient (Chinese Yuan*)	Total (n=8730)	ADHM cohort (n=1455)	UC cohort (n=7275)
Total costs	157249 ± 171122	157639 ± 119562	157170 ± 179673
Total costs in CHCs	34723 ± 95496	40143 ± 70922	33639 ± 99656
Total costs in hospitals†	122525 ± 137712	117496 ± 94575	123531 ± 144789
Total outpatient costs	38763 ± 78799	50956 ± 61235	36324 ± 81647
Outpatient costs in CHCs	18776 ± 63098	30090 ± 54804	16513 ± 64395
Outpatient costs in hospitals	19986 ± 44776	20866 ± 22969	19811 ± 47962
Total inpatient/ED costs	118486 ± 148831	106683 ± 100897	120846 ± 156566
Inpatient/ED costs in CHCs‡	15947 ± 73560	10053 ± 46545	17126 ± 77795
Inpatient/ED costs in hospitals	102539 ± 124346	96631 ± 87263	103720 ± 130477

* Average exchange rate in 2023: 1 Chinese Yuan=0·142 US Dollars.

† Hospitals refers to higher-level hospitals.

‡ A part of CHCs have emergency and inpatient ward.

ADHM= administrative-driven hierarchical management; CHC= community healthcare center; ED= emergency department; UC= usual care.

Supplementary Table 5. Eligibility criteria

Inclusion criteria
• Men or women aged ≥18 years<br>• With diagnosis of atrial fibrillation: the diagnosis must be identified from the Shanghai Municipal Health Commission database and verified by 12-lead electrocardiogram or Holter monitoring.<br>• Residents of Shanghai:<br>For the ADHM cohort: residents of Xuhui, Yangpu, or Changning district.<br>For the UC cohort: residents of Huangpu, Jing'an, Putuo, Hongkou, Pudong, Baoshan, Songjiang, Minhang, Jiading, Qingpu, Chongming, Fengxian, or Jinshan district.<br>• Taking part in the government-issued health insurance, including the Urban Residents' Basic Medical Insurance (URBMI), the Urban-Employ Based Medical Insurance (UEBMI) and the New Rural Cooperative Medical Scheme (NRCMS).<br>• For the ADHM cohort: willing to participate and able to sign informed consent.
Exclusion criteria
• Pregnant.<br>• Life expectancy <3 months.<br>• Requiring immediate hospitalization or already receiving inpatient care at the time of eligibility screening.

ADHM= administrative-driven hierarchical management; UC=usual care.

Supplementary Table 6. Implementation strategies of the ADHM model.

**Shanghai Municipal Health Commission**<br>• Launch of the White Paper on Standardized Management of Atrial Fibrillation in Shanghai<br>• Issuing administrative orders to the Health Commission of the pilot districts (Xuhui, Yangpu, and Changning districts) implementing the ADHM model.<br>• Semi-annual conferences on work<br>• Establishment of performance incentives for CHCs and GPs<br>• Access to the administrative port of the Shanghai AF Management Platform (hereinafter referred to as “the Platform”) for data monitoring and supervision
**District Health Commission**<br>• Issuing administrative orders to subordinate CHCs implementing the ADHM model.<br>• Implementation of performance incentives for CHCs<br>• Quarterly conference on work<br>• Ensuring the availability and stockpile of AF-related drugs in CHCs<br>• Access to the administrative port of the Platform for data monitoring and supervision<br>• Strengthening the regional healthcare network to facilitate referrals between subordinate CHCs and nearby higher-level hospitals
**Medical Association**[*]<br>• Guideline recommendation related to AF management<br>• Public health policy consultation<br>• Access to the administrative port of the Platform for data monitoring and supervision<br>• Knowledgebase updating for the Platform
**Shanghai AF Center Union**[#]<br>• Member hospitals of Shanghai AF Center Union accepting referrals from CHCs: interventional therapy, complications, and critical conditions<br>• Training for GPs on AF management<br>• Training for GPs on the use of the Platform

- Access to the administrative port of the Platform for data monitoring and supervision

CHCs

- Designation of at least one GP in charge of ADHM model implementation
- Implementation of performance and non-performance-based incentives for GPs. Note that the financial incentives are not tied to drug prescriptions or referrals to cardiac procedures but rather to data entry on the Shanghai AF management platform and regular patient follow-ups.
- Ensuring the availability and stockpile of AF-related drugs and examinations such as international normalized ratio
- Authorizing the installation of the Platform in the hospital network environment
- Access to the GP port of the Platform
- Organization of training conferences
- Organization of patient education activities

GPs

- Receiving training on AF management at the start of the intervention
- Receiving training on the use of the Platform at the start of the intervention
- Conferences every 6 months: sharing experience, discussing cases, and additional training on topics based on existing questions
- Access to the GP port of the Platform
- Enrollment of patients with AF
- Risk assessment: CHA_2DS_2-VASc, HAS-BLED, and EHRA scores
- Management: anticoagulation, symptom control, and management of cardiovascular risk factors, comorbidities, and complications, including hypertension, diabetes, coronary artery disease, chronic obstructive pulmonary disease, chronic kidney disease, etc.
- Follow-up: Follow-up plan, in-person follow-up, telephone follow-up
- Two-way referral: upward referral and receiving downward referral from upper-level hospitals

• Patient education: in-person education, push messages to the patient port through the Platform<br>• With the assistant of intelligent-generated, patient-individualized recommendations for treatment, follow-up plan and referral from the Platform<br>• Consultation with the specialist physician from the Shanghai AF Center Union for therapeutic decision-making if needed
**Patients with AF**<br>• Signing of the informed consent<br>• Access to the patient port of the Platform<br>• Receiving treatment including anticoagulation, symptom control, and management of cardiovascular risk factors, comorbidities, and complications<br>• Receiving push messages from the GPs regarding personalized AF-related risk scores, drug instruction, follow-up plan, and health education
**Family members of patients with AF**<br>• Access to the patient port of the Platform if authorized<br>• Receiving push messages from the GPs regarding personalized AF-related risk scores, drug instruction, follow-up plan, and health education

* including the Society of Cardiology of Shanghai Stroke Association, the Society of Cardiology of Shanghai Medical Association, the Society of Cardiology of Shanghai Physicians Association, and School of Public Health of Fudan University

# consist of secondary and tertiary hospitals

ADHM= administrative-driven hierarchical management; AF=atrial fibrillation; CHC=community healthcare center; GP=general practitioner

Supplementary Table 7. Characteristics before and after propensity score matching.

	Before propensity score matching				After propensity score matching			
Characteristics	ADHM cohort	control	SMD	P value	ADHM cohort	UC cohort	SMD	P value
Female sex (%)	45.9	51.7	0.097	<0.001	45.9	45.9	0.000	1.000
Age (years)	76.0	79.0	-0.247	<0.001	76.0	76.0	0.006	0.848
Hypertension (%)	92.0	95.7	0.097	<0.001	92.0	93.0	0.016	0.201
Diabetes (%)	54.6	50.6	0.079	0.002	54.6	54.5	0.003	0.908
Stroke/TIA (%)	51.4	55.4	-0.081	0.002	51.4	51.2	0.031	0.590
Systemic embolism (%)	3.9	4.6	-0.033	0.167	3.9	3.3	0.032	0.270
Peripheral artery disease (%)	4.3	3.5	0.044	0.096	4.3	3.4	0.048	0.091
Myocardial infarction (%)	3.4	5.9	-0.106	<0.001	3.4	3.0	0.021	0.471
Valvular heart disease (%)	3.5	3.0	0.027	0.305	3.5	3.3	0.014	0.630
COPD (%)	22.5	28.2	-0.126	<0.001	22.5	22.2	0.007	0.800
OSHAS (%)	1.1	0.6	0.061	0.021	1.1	0.7	0.042	0.144
Major bleeding (%)	11.9	16.4	-0.121	<0.001	11.9	11.0	0.029	0.308
History of cancer (%)	12.3	18.4	-0.159	<0.001	12.3	12.4	-0.004	0.896
Renal deficiency (%)	5.7	5.1	0.026	0.331	5.7	5.0	0.034	0.239
Liver deficiency (%)	15.9	15.8	0.002	0.929	15.9	16.1	-0.007	0.815

ADHM= administrative-driven hierarchical management; COPD= chronic obstructive pulmonary disease; OSAHS= Obstructive sleep apnea-hypopnea syndrome; SMD= standardized mean difference; TIA= transient ischemic attack; UC= usual care. The independent Student's t-tests and the $\chi 2$ tests were adopted for continuous and categorical variables, respectively. Two-sided P value was adopted.

Supplementary Table 8. Operation codes of cardiac interventions.

Cardiac interventions were screened by operation codes in the Shanghai Municipal Health Commission database.

Cardiac interventions	Codes of interventions
Ablation (radiofrequency, cryoballoon, surgical)	37.3401; 37.3302; 37.9903; 37.9904; 37.2600x001; 35.4200x008; 37.2300; 37.2600; 37.2100; 37.9900A; 89.5901; 37.3310HS; 37.3403; 37.3402; 37.2905; 37.3300x024; 37.3702; 37.3703; 37.3306
Left atrial appendage closure	37.9000x00; 37.9001; 88.5301
Cardioversion	99.6; 99.6201; 99.6100; 99.6200x001

Supplementary Table 9. Screening codes and definitions of outcome events.

Outcome events, including ischemic stroke, systemic embolism (SE), myocardial infarction (MI), major bleeding, acute heart failure (HF), were screened by ICD codes and were further excluded or verified by the medical records in the SHMHC database.

Outcomes	ICD-10 codes	Definitions during outcome verification
Ischemic stroke	I63, I64	- Neurological deficit of cerebrovascular cause that persists beyond 24 hours or is interrupted by death within 24 hours.<br>- Exclusion of coma due to severe brain trauma, intracranial tumor, metabolic disorder or fluid or electrolyte imbalance, peripheral neuropathy, or CNS infection.<br>- Not meeting criteria for subarachnoid hemorrhage, intraparenchymal hemorrhage and other hemorrhage<br>- With imaging findings
Systemic embolism (SE)	I74, K76.3, K28.0, D73.5, K55.0	- Acute vascular insufficiency or occlusion of the extremities or any non-CNS organ associated with clinical, imaging, surgical/autopsy evidence of arterial occlusion.<br>- Exclusion of other likely mechanism, e.g., trauma, atherosclerosis, or instrumentation, etc.
Myocardial infarction (MI)	I21, I22	- A rise of cardiac biomarker values (preferably cardiac troponin) with at least one value above the 99th percentile upper reference limit and with at least one of the following:<br>- Symptoms of ischemia<br>- ECG evidence: new ST-segment-T wave (ST-T) changes, or new left bundle branch block, or new pathological Q waves

		- Imaging evidence: new loss of viable myocardium or new regional wall motion abnormality<br>- Angiography evidence: intracoronary thrombus
Major bleeding	I60, I61, I62, I85.0, J47.x01, H35.6, K22.6, K25.0, K25.2, K25.4, K25.6, K26.0, K26.2, K26.4, K26.6, K27.0, K27.2, K27.4, K27.6, K28.0, K28.2, K28.4, K28.6, K66.1, K76.801, K76.803, K76.811, K76.814, K92.0, K92.1, K92.2, R04.2, R04.8, R04.9, R57.1, R58.x01	At least one of the following conditions:<br>- Fatal bleeding<br>- Symptomatic bleeding in a critical organ, such as intracranial, intraspinal, intraocular, retroperitoneal, intra-articular or pericardial, or intramuscular with compartment syndrome<br>- Bleeding causing a fall in hemoglobin level of 20 g/L or more or leading to transfusion of two or more units of whole blood or red cells.
Acute Heart failure (HF)	I50.101, I50.103, I50.104, I50.907	- Hospitalization, or emergency department visit requiring treatment with infusion therapy, for a clinical syndrome that presents with multiple signs and symptoms consistent with cardiac decompensation or inadequate cardiac pump function.<br>- Evidence in the doctor’s notes that the reason for this hospitalization, or emergency department visit, was heart failure.<br>- Evidence of signs and symptoms: increasing or new onset shortness of breath, edema, paroxysmal nocturnal dyspnea, orthopnea, and hypoxia<br>- Include acute decompensated HF with

		preserved, mid-range, and reduced left ventricular ejection fraction.<br>- If a patient was diagnosed and treated for HF in an outpatient setting without infusion therapy for decompensated HF, such condition was not considered an outcome.

CNS= central nervous system; SHMHC= Shanghai Municipal Health Commission

Supplementary Table 10. Classification of death.

The death and causes of death were obtained from the database of Shanghai Municipal Center for Disease Control & Prevention.

**Cardiovascular death**
• Ischemic heart diseases<br>• Hypertensive heart diseases<br>• Other heart and vascular diseases, including myocarditis, endocarditis, pulmonary embolism, aortic aneurysm, dissecting aneurysm<br>• Sudden, unexpected, and unwitnessed death or death of unknown cause<br>• Ischemic stroke<br>• Hemorrhagic stroke and non-traumatic intracranial bleeding
**Non-cardiovascular death**
• Alzheimer's disease, Parkinson's disease, other neurological diseases except vascular central nervous system causes<br>• Diabetes mellitus<br>• Cancers (malignant solid tumors)<br>• Chronic obstructive pulmonary disease, lower respiratory infections, and other respiratory diseases<br>• Gastrointestinal and liver diseases<br>• Chronic kidney diseases and other kidney diseases<br>• Leukemia, lymphoma, and other diseases of blood system<br>• Falls<br>• Road injuries<br>• Drowning and asphyxia.<br>• Self-harm<br>• COVID-19<br>• Other non-cardiovascular causes of death.

Supplementary methods

Shanghai AF Management Platform

The Shanghai Atrial Fibrillation (AF) Management Platform was designed to facilitate the implementation of the ACC-to-ABC pathway in the administrative-driven hierarchical management (ADHM) cohort. The electronic platform included three ports, i.e., the general practitioner (GP) port, the patient port, and the administrative port, facilitating GPs' management, patient education and administrative supervision, respectively.

The GP port consisted of 5 modules. Module 1 (Risk Assessment): the GPs were required to collect the demographic information, symptoms and signs, comorbidities and complications (including hypertension, diabetes mellitus, chronic obstructive pulmonary disease [COPD], and history of stroke, myocardial infarction [MI] and cancer, etc.), prior treatment process (including drugs, catheter ablation and left atrial appendage closure [LAAC], etc.), and generate the EHRA, CHA2DS2-VASc and HAS-BLED scores to assess the AF-related symptoms, stroke, and bleeding risks. Module 2 (Treatment): the GPs were required to customize a treatment plan, including stroke prevention, symptom control and management of comorbidities and complications based on the information in the Module 1. Module 3 (Follow-up): the GPs were required to customize a follow-up plan (such as every three months) based on the information in the first 2 modules. During every follow-up visit, the GPs evaluate adverse events, the compliance of the current therapy, followed by risk score reevaluation and treatment plan readjustment. Module 4 (Referral): the GPs generated referral suggestions for interventional therapy or complication handling when necessary. The referral module of the Shanghai AF Management Platform provided a prompt if a patient met the criteria for referral as regulated by the White Paper on the Standardized Prevention and Treatment of Atrial Fibrillation in Shanghai. Module 5 (Advice): based on the information inputted in Module 1-4, the platform automatically generated medical advice in accordance with the Chinese guideline in pop-up windows. For example, if a patient presented with a EHRA level 2b, CHA2DS2-VASc score 5, and HAS-BLED score 2, the generated advice would be a recommendation of anticoagulant prescription with relatively mild concern about bleeding risks, and potential referral for catheter ablation. However, the final therapeutic or referral decisions were made by the

GPs.

In the patient port, the participants in the ADHM cohort would receive short messages regarding AF-related health education, drug instructions and personized follow-up plan regularly pushed from the GP port. The authorized family members would also receive the same messages, aiming to provide family support.

The administrative port was accessed by the Shanghai Municipal Health Commission and authorized researchers, providing panoramic view, data monitoring, statistical analyses, authority control, prescription oversight, and knowledgebase updating, etc. The administrative port supervised the data inputted from the GP port and might contact the GPs if deviation from the protocol occurred.
