# Supplementary material for: Administrative-driven hierarchical management of atrial fibrillation on cardiovascular events: a prospective matched cohort study
Source: Nat Commun. 2025 Dec 16;16:11395. doi: 10.1038/s41467-025-66203-y (PMC12738700; doi:10.1038/s41467-025-66203-y)
Supplement: Supplementary file 2 — Reporting Summary [file 41467_2025_66203_MOESM2_ESM.pdf]

nature portfolio

Corresponding author(s): Yi-Gang Li, Qunshan Wang

Last updated by author(s): Apr 30, 2025

Reporting Summary

Nature Portfolio wishes to improve the reproducibility of the work that we publish. This form provides structure for consistency and transparency in reporting. For further information on Nature Portfolio policies, see our Editorial Policies and the Editorial Policy Checklist.

Statistics

For all statistical analyses, confirm that the following items are present in the figure legend, table legend, main text, or Methods section.

n/a	Confirmed	
☐	☒	The exact sample size (*n*) for each experimental group/condition, given as a discrete number and unit of measurement
☐	☒	A statement on whether measurements were taken from distinct samples or whether the same sample was measured repeatedly
☐	☒	The statistical test(s) used AND whether they are one- or two-sided<br>*Only common tests should be described solely by name; describe more complex techniques in the Methods section.*
☐	☒	A description of all covariates tested
☐	☒	A description of any assumptions or corrections, such as tests of normality and adjustment for multiple comparisons
☐	☒	A full description of the statistical parameters including central tendency (e.g. means) or other basic estimates (e.g. regression coefficient) AND variation (e.g. standard deviation) or associated estimates of uncertainty (e.g. confidence intervals)
☐	☒	For null hypothesis testing, the test statistic (e.g. *F*, *t*, *r*) with confidence intervals, effect sizes, degrees of freedom and *P* value noted<br>*Give P values as exact values whenever suitable.*
☒	☐	For Bayesian analysis, information on the choice of priors and Markov chain Monte Carlo settings
☐	☒	For hierarchical and complex designs, identification of the appropriate level for tests and full reporting of outcomes
☒	☐	Estimates of effect sizes (e.g. Cohen's *d*, Pearson's *r*), indicating how they were calculated

Our web collection on statistics for biologists contains articles on many of the points above.

Software and code

Policy information about availability of computer code

Data collection	Data collection were performed using Python Version 3.12.0 (Python Software Foundation, OR, USA). The code that supports the findings are available at 10.5281/zenodo.17012225.
Data analysis	Statistical analyses were performed using SPSS Version 27.0 (IBM Corp., NY, USA) and Prism 9 (GraphPad Software, CA, USA).

For manuscripts utilizing custom algorithms or software that are central to the research but not yet described in published literature, software must be made available to editors and reviewers. We strongly encourage code deposition in a community repository (e.g. GitHub). See the Nature Portfolio guidelines for submitting code & software for further information.

Data

Policy information about availability of data

All manuscripts must include a data availability statement. This statement should provide the following information, where applicable:

- Accession codes, unique identifiers, or web links for publicly available datasets
- A description of any restrictions on data availability
- For clinical datasets or third party data, please ensure that the statement adheres to our policy

The data generated in this study are provided in the Source Data file. Further data can be requested from the corresponding authors. Requests for data will require the approval from the SHMHC and a data access agreement to be signed.

Research involving human participants, their data, or biological material

Policy information about studies with human participants or human data. See also policy information about sex, gender (identity/presentation), and sexual orientation and race, ethnicity and racism.

Reporting on sex and gender	We used sex throughout the entire manuscript. Sex was based on Chinese ID. Both sexes were included in this study. During the design of the study, both sexes were included. No individual-level data was shared. In this study, we included 4008 (45.9%) females and 4722 (54.1%) males. We did not perform sex-based analyses as the study protocol did not include pre-specified sex- or gender-based analyses.
Reporting on race, ethnicity, or other socially relevant groupings	The study was conducted in China. All participants were Chinese.
Population characteristics	We enrolled patients with a diagnosis of AF, aged over 18 years, and residents of Shanghai with government-issued medical insurance. Exclusion criteria included subjects with a life expectancy of less than three months, pregnant, or patients requiring immediate hospitalization or already receiving inpatient care. The characteristics of the population were presented in Table 1.
Recruitment	The study consisted of an intervention group (ADHM cohort) and a matched control group (UC cohort). The flowchart was shown in Supplementary Fig 4. Inclusion criteria were as follows: patients with a diagnosis of AF, aged over 18 years, and residents of Shanghai with government-issued medical insurance. Exclusion criteria included subjects with a life expectancy of less than three months, pregnant, or patients requiring immediate hospitalization or already receiving inpatient care. Detailed eligibility criteria are included in the Supplementary Table 5.
Ethics oversight	The study was conducted in accordance with the declaration of Helsinki. The study was approved by the institutional review board of Xinhua Hospital, School of Medicine, Shanghai Jiao Tong University, China, and registered with chictr.org.cn, Chinese Clinical Trial Registry (ChiCTR2000036931). The study was also authorized by the SHMHC through the administrative documents. Written informed consent was obtained from participants from the ADHM cohort before any study related procedures.

Note that full information on the approval of the study protocol must also be provided in the manuscript.

Field-specific reporting

Please select the one below that is the best fit for your research. If you are not sure, read the appropriate sections before making your selection.

- [x] Life sciences
- [ ] Behavioural & social sciences
- [ ] Ecological, evolutionary & environmental sciences

For a reference copy of the document with all sections, see nature.com/documents/nr-reporting-summary-flat.pdf

Life sciences study design

All studies must disclose on these points even when the disclosure is negative.

Sample size	The ADHM cohort enrolled 1455 patients from 34 CHCs in the designated districts. The UC cohort were matched at 1:5 ratio. The sample size was not calculated, however, with the government authority, we enrolled all AF patients who admitted to CHCs between August 1 and November 28, 2022.
Data exclusions	No collected data were excluded.
Replication	We constructed different UC cohort using different methods of matching in the initial version and revised version of the manuscript. The results were replicable. The propensity score matching was performed to control the covariates.
Randomization	It was not a randomized study. However, we performed propensity score matching to minimize the confounding factors.
Blinding	Blinding is not possible in this study since patients were under different management, either by GP or self management.

Reporting for specific materials, systems and methods

We require information from authors about some types of materials, experimental systems and methods used in many studies. Here, indicate whether each material, system or method listed is relevant to your study. If you are not sure if a list item applies to your research, read the appropriate section before selecting a response.

Materials & experimental systems

n/a	Involved in the study
☒	☐ Antibodies
☒	☐ Eukaryotic cell lines
☒	☐ Palaeontology and archaeology
☒	☐ Animals and other organisms
☐	☒ Clinical data
☒	☐ Dual use research of concern
☒	☐ Plants

Methods

n/a	Involved in the study
☒	☐ ChIP-seq
☒	☐ Flow cytometry
☒	☐ MRI-based neuroimaging

Clinical data

Policy information about clinical studies
All manuscripts should comply with the ICMJE guidelines for publication of clinical research and a completed CONSORT checklist must be included with all submissions.

Clinical trial registration	the study was registered with chictr.org.cn, Chinese Clinical Trial Registry (ChiCTR2000036931).
Study protocol	The study protocol was available from the corresponding authors upon reasonable request.
Data collection	Between August 1 and November 28, 2022, a total of 8730 patients with AF were included in the study, including 1455 in the ADHM cohort and 7275 in the matched usual care (UC) cohort. Patients in the ADHM cohort were enrolled from 34 CHCs in the pilot districts, including 543 patients from 12 CHCs in Xuhui District, 531 from 12 CHCs in Yangpu District, and 381 from 10 CHCs in Changning District. Patients in the UC cohort were residents of the other 13 districts of Shanghai.
Outcomes	The primary outcome was the composite of CV death, ischemic stroke, SE, MI, major bleeding, and acute HF. The secondary outcome was set as the components of the primary outcomes, all-cause death, and non-CV death, at 30 months.

Plants

Seed stocks	This study did not involve plants.
Novel plant genotypes	This study did not involve plants.
Authentication	This study did not involve plants.
